# Environmental‐relevant bisphenol A exposure promotes ovarian cancer stemness by regulating microRNA biogenesis

**DOI:** 10.1111/jcmm.17920

**Published:** 2023-08-23

**Authors:** Sophia S. N. Lam, Zeyu Shi, Carman K. M. Ip, Chris K. C. Wong, Alice S. T. Wong

**Affiliations:** ^1^ School of Biological Sciences University of Hong Kong Hong Kong China; ^2^ Laboratory for Synthetic Chemistry and Chemical Biology Limited Hong Kong Science and Technology Parks Hong Kong China; ^3^ Cellular Screening Center University of Chicago Chicago Illinois USA; ^4^ Department of Biology Hong Kong Baptist University Hong Kong China

**Keywords:** bisphenol A, cancer stem cells, microRNA, oestrogen receptors, ovarian cancer

## Abstract

Bisphenol A (BPA) is a ubiquitous environmental xenobiotic impacting millions of people worldwide. BPA has long been proposed to promote ovarian carcinogenesis, but the detrimental mechanistic target remains unclear. Cancer stem cells (CSCs) are considered as the trigger of tumour initiation and progression. Here, we show for the first time that nanomolar (environmentally relevant) concentration of BPA can markedly increase the formation and expansion of ovarian CSCs concomitant. This effect is observed in both oestrogen receptor (ER)‐positive and ER‐defective ovarian cancer cells, suggesting that is independent of the classical ERs. Rather, the signal is mediated through alternative ER G‐protein‐coupled receptor 30 (GPR30), but not oestrogen‐related receptor α and γ. Moreover, we report a novel role of BPA in the regulation of Exportin‐5 that led to dysregulation of microRNA biogenesis through miR‐21. The use of GPR30 siRNA or antagonist to inhibit GPR30 expression or activity, respectively, resulted in significant inhibition of ovarian CSCs. Similarly, the CSCs phenotype can be reversed by expression of Exportin‐5 siRNA. These results identify for the first time non‐classical ER and microRNA dysregulation as novel mediators of low, physiological levels of BPA function in CSCs that may underlie its significant tumour‐promoting properties in ovarian cancer.

## 1 INTRODUCTION

Bisphenol A (BPA) contamination is widespread with a global consumption of over million metric tons yearly.^1^, ^2^ Despite the proclaimed short half‐life and high metabolic clearance rate of BPA in the human body, there is considerable evidence that the biological effects of BPA can still be seen with chronic exposure at low doses in daily lives.^3^ Indeed, nearly 93% of the people tested have detectable BPA in their systems (0.2–18.9 ng/mL), which infants and children have the highest level.^3^ A survey of the environmental exposure for seven phenols on 2256 people reported that BPA was found in almost all samples with a geometric mean concentration of 1.905 μg/L and a maximum urinary concentration of 399 μg/L.^4^ The US Centers for Disease Control and Prevention (CDC) identified BPA from over 2460 people that BPA exposure is widespread, and the geometric mean urine concentration was calculated to be 1.9 μg//L.^5^ However, its effect has only been characterized without precluding external contamination. The reproductive system is a specific target organ of BPA. Prenatal BPA exposure can result in numerous ovarian abnormalities and alter oocyte meiotic events, even at doses below the reference dose.^6^ There also exists a positive correlation between BPA exposure and increased incidence of polycystic ovarian syndrome and infertility.^7^, ^8^ BPA alters gene expression inducing the growth and dissemination of cultured ovarian cancer cells.^9^, ^10^, ^11^ However, how the low BPA exposure exerts its carcinogenic action remains unclear.

Given its structural similarity to oestrogen, BPA's oestrogenic action has long been suspected. However, BPA binds to ERα at a 10,000‐fold lower affinity than that of oestrogen.^12^ There is also evidence that BPA can act through nonclassical ERs, including oestrogen‐related receptors (ERRs)^13^ and G‐protein‐coupled receptor 30 (GPR30).^14^ For example, ERRs are frequently expressed in ovarian carcinomas and associated with poor overall survival in serous ovarian cancer patients.^15^ It is reported that activated GPR30 can enhance ER‐negative breast cancer cell proliferation.^16^, ^17^

It is becoming clear that a minute population of cancer stem cells (CSCs) in the bulk tumour is crucial for carcinogenesis. CSCs possess the ability to self‐renew and differentiate to give rise to other subpopulations. These cells also display a greatly enhanced tumour‐initiating capability and are responsible for tumour recurrence and metastasis.^18^ Therefore, CSC survival selection advantage could be of critical importance to all stages of carcinogenesis. There is a need to better understand the environmental signals and regulatory pathways contributing to CSC formation or expansion to circumvent tumour development and progression.

In this study, we show for the first time that the environmental‐relevant, low doses of BPA is already adequate to accelerate ovarian CSC formation and expansion. We also provide evidence that this is via non‐classical ER GPR30 through miRNA biogenesis dysregulation.

## 2 MATERIALS AND METHODS

### 2.1 Chemicals and cell culture

BPA obtained from Sigma was dissolved in DMSO. G15 were purchased form Cayman Chemical. Human ovarian cancer cell lines, HEY‐A8 and SKOV‐3 (generous gifts from Dr. J. Liu at MD Anderson Cancer Center, and Dr. N. Auersperg at University of British Columbia) were cultured in phenol‐red free medium‐199 (Sigma) supplemented with 5% charcoal and dextran treated FBS, 100 U/mL penicillin and streptomycin (Invitrogen) in a humidified incubator with 5% CO_2_ at 37°C. Cell lines used were verified using AmpFLSTR Identifier Plus PCR Amplification Kit (Invitrogen), and data were analysed by GeneMapper 4.1 Software.

### 2.2 Small interfering RNA and miR inhibitor mediated gene knockdown

ERRα, ERRγ, GPR30, Exportin‐5 specific siRNA duplexes and nonspecific siRNA oligonucleotides were obtained from Dharmacon. Transfection was achieved at 20 nmol/L working concentration using siLentFect (Bio‐Rad) according to the instructions. In short, Opti‐MEM (Gibco) and siLenFect (Bio‐Rad) mixture and Opti‐MEM and siRNA mixture were prepared, and incubated for 5 min. The diluted siRNA was added to the diluted siLenFect. Mix by pipetting and incubated for 20 min before dropping into well with replaced medium. Anti‐miR‐21 (Anti‐miRNA™ miRNA inhibitor miR‐21, Cat. no.: AM17000) and non‐specific miR (Anti‐miRNA™ inhibitor Negative Control #1, Cat. no.: AM17010) were purchased from ThermoFisher. Anti‐miR‐21 and NS miR were transfected into cells at 20 nM concentration using Lipofectamine RNAiMAX reagent according to the protocol.

### 2.3 Reverse transcription PCR analysis

Total RNA was extracted using Trizol reagent (Invitrogen). M‐MLV reverse transcriptase (Promega BioSciences) were used to reverse transcription following instructions. In brief, Oligo (dT), 1 μg mRNA, dNTPs and DEPC water were mixed in a total volume of 12 μL. 5◊first‐strand buffer, 0.1 M DTT and RNase OUT™ recombinant ribonuclease inhibitor (Invitrogen) were added and incubated at 37°C for 2 min. M‐MLV‐RT were then added and incubated at 37°C for 50 min and 70°C for 15 min. miR‐21, miR‐93 and internal loading control U6 were reverse transcribed by Applied Biosystems™ TaqMan™ MicroRNA Reverse Transcription Kit (ThermoScientific) using stem loop primers 5′‐GTT GGC TCT GGT GCA GGG TCC GAG GTA TTC GCA CCA GAG CCA AGC AAC A‐3′ (miR‐21), 5′‐GTT GGC TCT GGT GCA GGG TCC GAG GTA TTC GCA CCA GAG C CA ACT ACC T‐3′(miR‐93) and 5’‐GTC GTA TCC AGT GCA GGG TCC GAG GTA TTC GCA CTG GAT ACG ACA AAA ATA T‐3′ (U6). Shortly, the RT master mix containing dNTP, Multiscribe™ RT enzyme, RT Buffer, RNase Inhibitor and nuclease free water were prepared to a total volume of 7 μL and mixed gently. Total RNA with the RT master mix were combined in a ratio of 5:7 in tube containing 3 μL RT primers and GoTaq® G2 Flexi DNA Polymerase (Promega, Madison, WI). PCR was performed at 16°C for 30 min, 42°C for 30 min, 85°C for 5 min using Bio‐Rad C1000 Thermal Cycler (Bio‐Rad). Gel images were visualized on Gel Doc XR+ Gel Documentation System (Bio‐Rad). Gene‐specific primers used were: 5’‐TGC CAA TTC AGA CTC TGT GC‐3′ (ERRα forward); 5’‐TCT CCA AGT CCC ACT CTG CT‐3′ (ERRα reverse); 5’‐CAT GGA GCA CTG TCC TCA GA‐3′ (ERRβ forward); 5′‐AGT TGC AAG CCA AGC TCA TT‐3′ (ERRβ reverse); 5’‐CAG CTG TTC GTC CTT CAT CA‐3′ (ERRγ forward); 5′‐GTG GTA CCC AGA AGC GAT GT‐3′ (ERRγ reverse); 5′‐GTT CAG CAG TGC CGT GTA GA‐3′ (GPR30 forward); 5’‐TCT GTG TGA GGA GTG CAA GG‐3′ (GPR30 reverse); 5′‐ATG TGT GTG CTT TGT GGA G‐3′ (BMI‐1 forward); 5′‐AGT GGT CTG GTC TTG TGA AC‐3′ (BMI‐1 reverse); 5′‐AAG ACA AGG TCC CGG TCA AG‐3′ (Nanog forward); 5′‐CCT AGT GGT CTG CTG TAT TAC‐3′ (Nanog reverse); 5’‐TCA TGG TCG GAT CAC AAA GA‐3′ (c‐Kit forward); 5′‐AGG GGC TGC TTC CTA AAG AG‐3′ (c‐Kit reverse); 5′‐ GAT CTG GAA GTC GCT CCC CAA G‐3′ (Drosha forward); 5′‐ATG GTC TCC TCG GGC TCT TT‐3′ (Drosha reverse); 5′‐GTG CTG CAG TAA GCT GTG CTA‐3′ (Dicer forward); 5’‐TGC TGA AGT CTC CCC TGA TCT‐3′ (Dicer reverse); 5’‐CGA GCA CAA CAA GGA GAG GT‐3′ (Exportin‐5 forward); 5′‐GGT TCT GGG GGC CTT ACT TT‐3′ (Exportin‐5 reverse); 5′‐TCA CCG AGG CCC CTC TGA ACC CTA GA‐3′; (β‐actin forward); 5′‐GGC AGT AAT CTC CTT CTG CAT CCT‐3′ (β‐actin reverse); 5′‐TAG CTT ATC AGA CTG ATG TTG A‐3′ (miR‐21 forward); 5’‐CAA AGT GCT GTT CGT GCA GGT AG‐3′ (miR‐93 forward); 5’‐GCA AGG ATG ACA CGC AAA T‐3′ (U6 forward); 5’‐TGC AGG GTC CGA GGT ATT CG‐3′ (Universal reverse for miRNA); 5′‐GTG CAG GGT CCG AGG T‐3′ (U6 reverse).

### 2.4 Sphere formation assay

CSCs were enriched in suspension culture as previously described.^19^ In brief, ovarian cancer cells were trypsinized and resuspended in non‐adherent petri dish coated with 5 mL 0.5% agarose (Invitrogen) to avoid cell adhesion. After 72 h, spheroids were photographed using Olympus CKX53 Compact Cell Culture Microscope (Olympus). Spheroids with diameter greater or equal to 100 μm were counted.

### 2.5 MTT analysis

Proliferation rates were evaluated by the colorimetric MTT (3‐(4,5‐dimethylthiazol‐2‐yl)‐2,5‐diphenyltetrazolium) bromide assay (Sigma). Briefly, 10% MTT solution were added in the culture medium then the microplate was incubated for 1 h at 37°C. Insoluble formazans were resuspended in 100 μL DMSO. A microplate reader (Bio‐Rad) was used to perform colorimetric analysis at 570 nm wavelength.

### 2.6 Statistical analysis

Results are analysed with unpaired *t‐*test (GraphPad) or two‐way anova and showed as the mean ± SD. *p* < 0.05 was considered as statistically significant.

## 3 RESULTS

### 3.1 BPA drives tumour‐initiation and self‐renewal of ovarian CSCs

**FIGURE 1:**
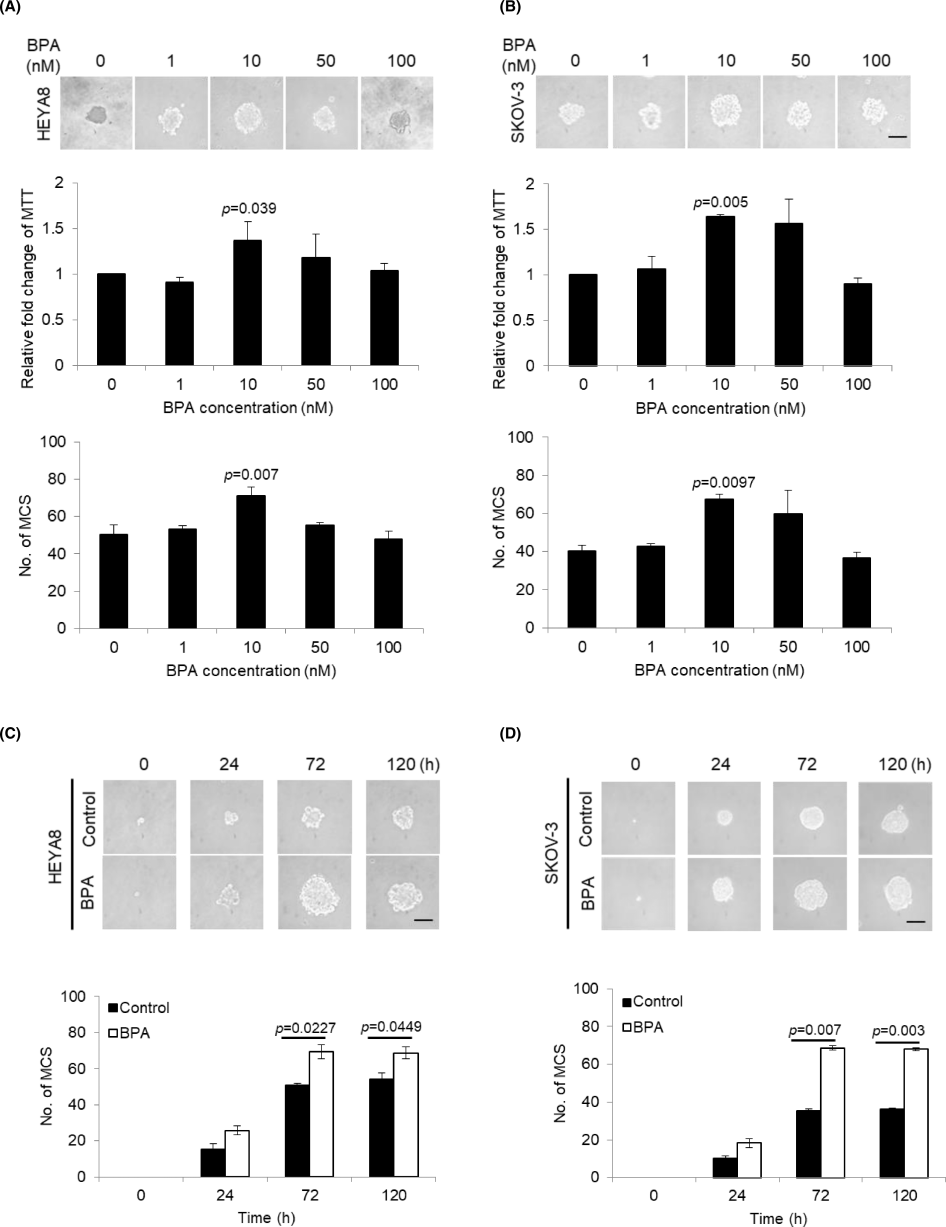
BPA stimulates MCS formation at low concentration. (A) HEYA8 and (B) SKOV‐3 were cultured with BPA at 0, 10, 50 and 100 nM in non‐adherent culture dish for 72 h. The number of tumour spheres generated was photographed, quantified by MTT assay and counted. (C) HEYA8 and (D) SKOV‐3 were cultured with BPA at 0 or 10 nM in non‐adherent culture dish for 0, 24, 72 and 120 h. The number of tumour spheres generated was photographed and counted. Bar = 100 μm. Experiments were repeated three times and results are presented as the mean ± SD and were analysed using unpaired student's *t* test.

The ability to form spheres in nonadherent cultures in serum‐free medium is one of the characteristics of CSCs.^19^ We first examined the effects of BPA on CSC sphere growth. Figure 1 shows that ovarian cancer cells treated with environmentally related low doses of BPA could cause a significant enhancement in sphere formation and growth in a dose‐dependent manner, with maximum change observed at 10 nM. This dose was close to that of the environmentally relevant BPA^4^, ^5^ The response to higher doses of BPA (50–100 nM) was insignificant (Figure 1A,B). DMSO was included as control (0 nM).

**FIGURE 2:**
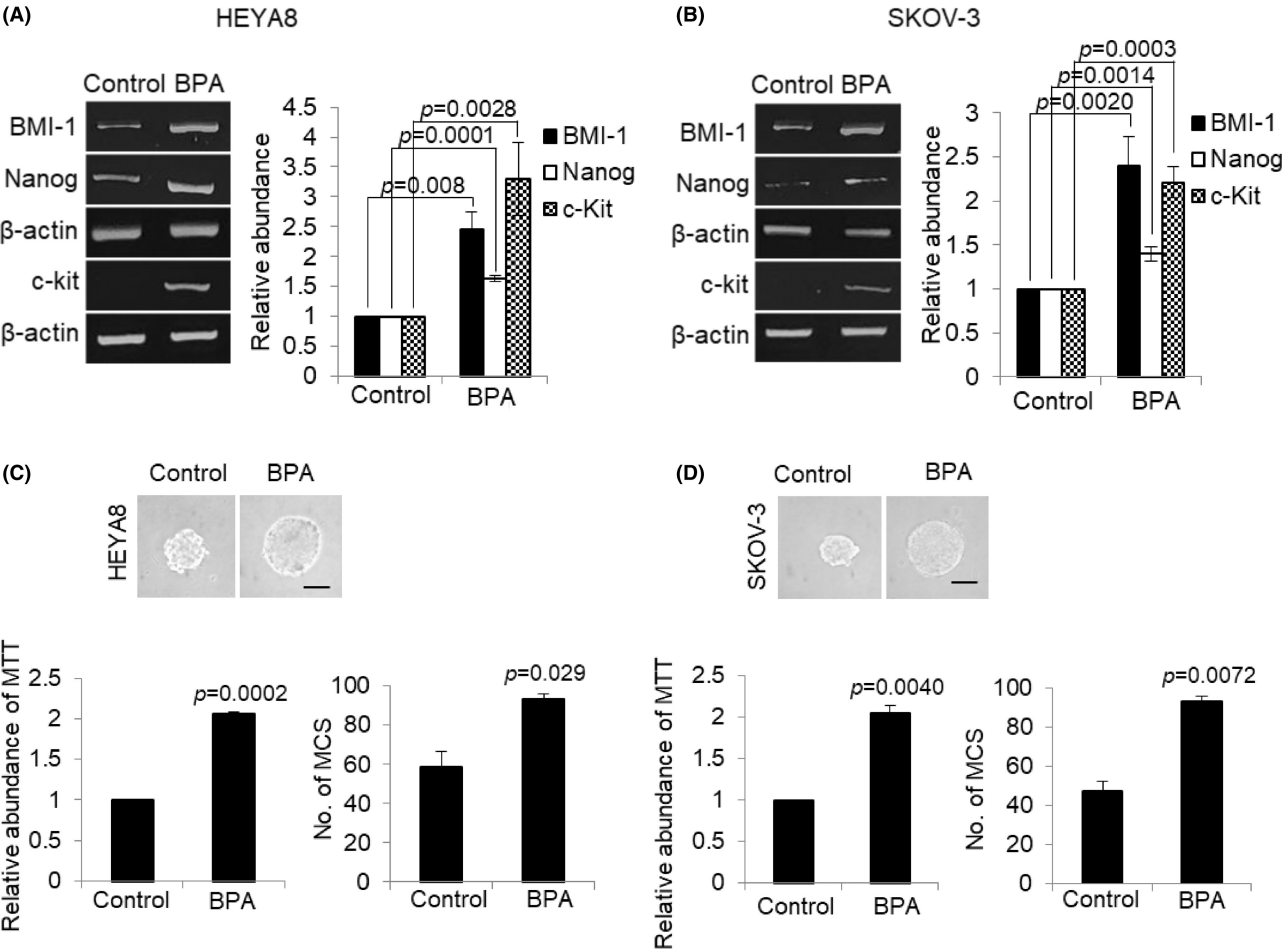
BPA induces cancer stem cell phenotypes. (A) HEYA8 and (B) SKOV‐3 were cultured with BPA at 0 and 10 nM in non‐adherent culture dish for 72 h. Total RNA was extracted and reverse transcription‐PCR was performed using BMI‐1, Nanog, and c‐Kit sequence‐specific primers. The signal intensities were quantified by densitometric analysis and the amount was normalized for the amount of β‐Actin. (C) HEYA8 and (D) SKOV‐3 were cultured with BPA at 0 or 10 nM in non‐adherent culture dish for 72 h. After 72 h, tumour spheres were recovered, dissociated and incubated for another 72 h for secondary sphere formation. The number of tumour spheres generated was photographed, quantified by MTT assay and counted. Bar = 100 μm. Experiments were repeated three times and results are presented as the mean ± SD and were analysed using unpaired student's *t* test.

Furthermore, this increase was also observed at different time points with maximal increase at 72 h (Figure 1C,D). To more precisely quantify the CSC‐like phenotype, we assessed the expression of stemness markers. Using RT‐PCR, our results revealed high and widespread expression of BMI‐1, Nanog, and CD117 (c‐Kit) in BPA‐treated cells, suggesting that BPA accelerates the transition to stem‐like phenotype (Figure 2A,B). Since spheroid formation capabilities in serial passages is an indirect indicator in support of stem cell self‐renewal,^20^ we further verified the effect of BPA in recurrence experiment. Primary spheres treated with 10 nM BPA were dissociated and replated to form secondary spheres. The growth of the secondary sphere assessed by MTT showed a more than two‐fold increase in BPA‐induced growth (Figure 2C). The capacity of the secondary sphere formation was also significantly higher in BPA‐treated cells compared with controls (~1.6‐fold and ~2‐fold, respectively) (Figure 2D), suggesting an enhanced self‐renewal capacity.

### 3.2 GPR30 is a target of BPA‐mediated CSC regulation

**FIGURE 3:**
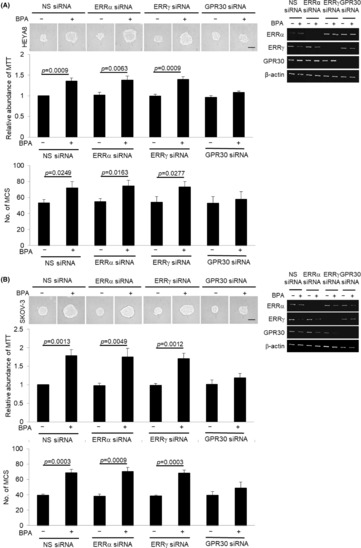
BPA induces MCS formation via GPR30. (A) HEYA8 and (B) SKOV‐3 transfected with nonspecific (NS) siRNA, ERRα siRNA ERRγ siRNA or GPR30 siRNA were cultured with BPA at 0 or 10 nM in non‐adherent culture dish for 72 h. The number of tumour spheres generated was photographed, quantified by MTT assay and counted. Total RNA was extracted and reverse transcription‐PCR was performed using ERα, ERβ, ERRα, ERRβ, ERRγ and GPR30 sequence‐specific primers. Bar = 100 μm. Experiments were repeated three times and results are presented as the mean ± SD and were analysed using unpaired student's *t* test.

Since SKOV‐3 which has defective ERα receptor also demonstrated similar BPA‐induced sphere formation as in HEYA8 which consists of functional ERα receptor, we hypothesized that BPA may induce the growth of CSCs and sphere formation in an ER‐independent manner through alternative receptors, which are ERRα, ERRγ and GPR30, that have key roles in ovarian cancer progression.^21^ Although ERRα, ERRγ, and GPR30 were all expressed on HEYA8 and SKOV‐3, only silencing of GPR30 with siRNA abrogated BPA‐mediated sphere formation and growth (Figure 3A,B) while nonspecific siRNA had no effect. In contrast, blockade of ERRα or ERRγ, which were also present on cells, did not produce any inhibition on the BPA‐mediated sphere formation (Figure 3A,B), suggesting a key role of GPR30 in BPA‐mediated CSC regulation.

### 3.3 BPA increases Exportin‐5 expression in CSCs

**FIGURE 4:**
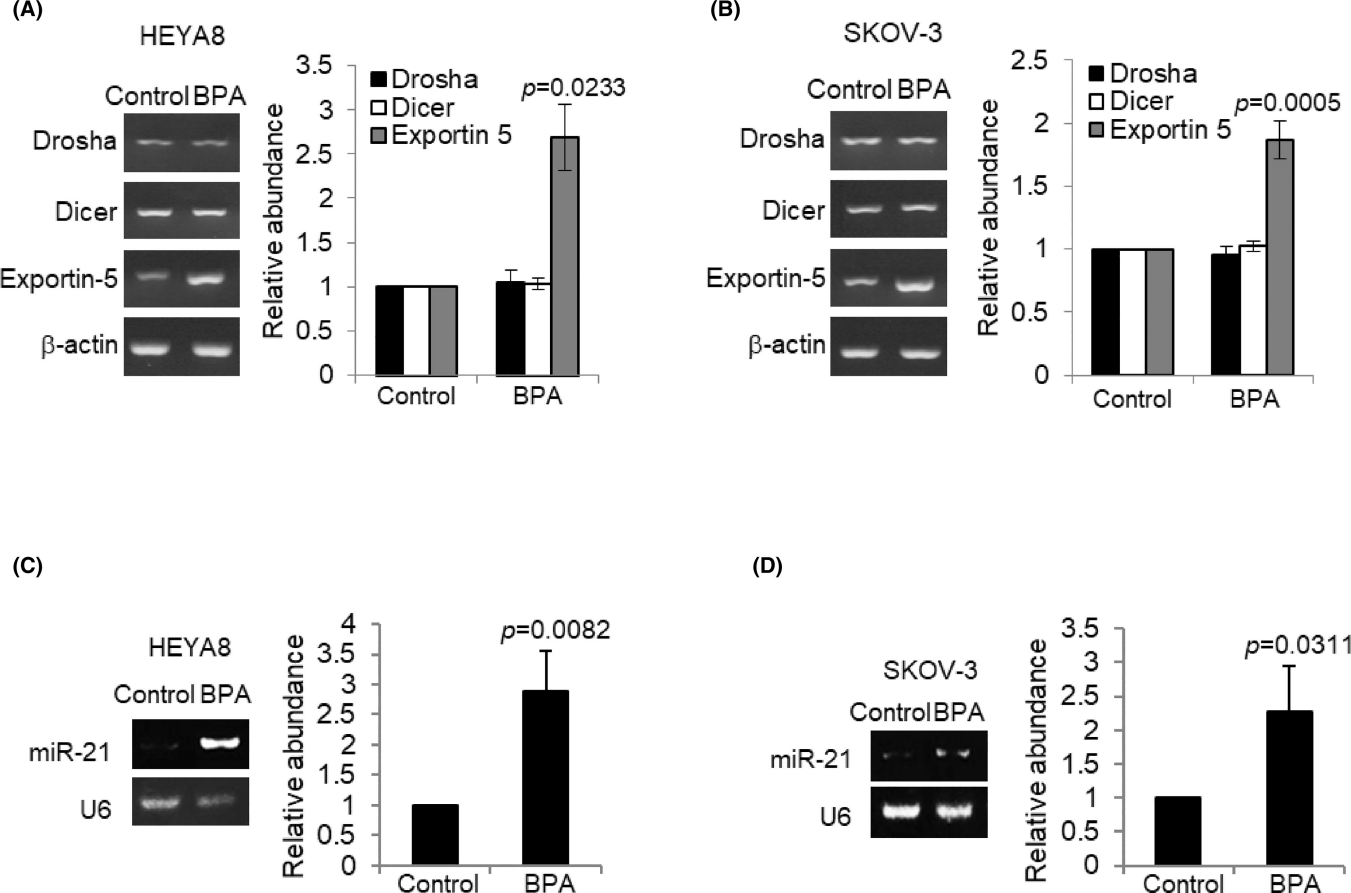
BPA induces Exportin‐5 and miR‐21 expression. (A) HEYA8 and (B) SKOV‐3 were cultured with BPA at 0 and 10 nM in non‐adherent culture dish for 72 h. Total RNA was extracted and reverse transcription‐PCR was performed using Drosha, Dicer, and Exportin‐5 sequence‐specific primers. The signal intensities were quantified by densitometric analysis and the amount was normalized for the amount of β‐Actin. (C) HEYA8 and (D) SKOV‐3 were cultured with BPA at 0 and 10 nM in non‐adherent culture dish for 72 h. Total RNA was extracted and reverse transcription‐PCR was performed using miR‐21 sequence‐specific primer. The signal intensities were quantified by densitometric analysis and the amount was normalized for the amount of U6. Experiments were repeated three times and results are presented as the mean ± SD and were analysed using unpaired student's *t* test.

Next, we investigated the mechanism by which BPA could regulate the CSC phenotype. There is a strong correlation between Dicer/Drosha suppression and more aggressive tumour phenotype such as stemness.^22^ We, therefore, examined whether BPA may regulate Dicer/Drosha expression in ovarian CSCs. However, the expression of Dicer/Drosha remained unchanged (Figure 4A,B). Additional mechanisms are also possible, such as the Exportin‐5, which has been implicated in the nuclear export of pre‐miRNA.^7^ We found a significant positive correlation between BPA and the expression of Exportin‐5 (Figure 4A,B).

### 3.4 Exportin‐5 expression induces miR‐21

**FIGURE 5:**
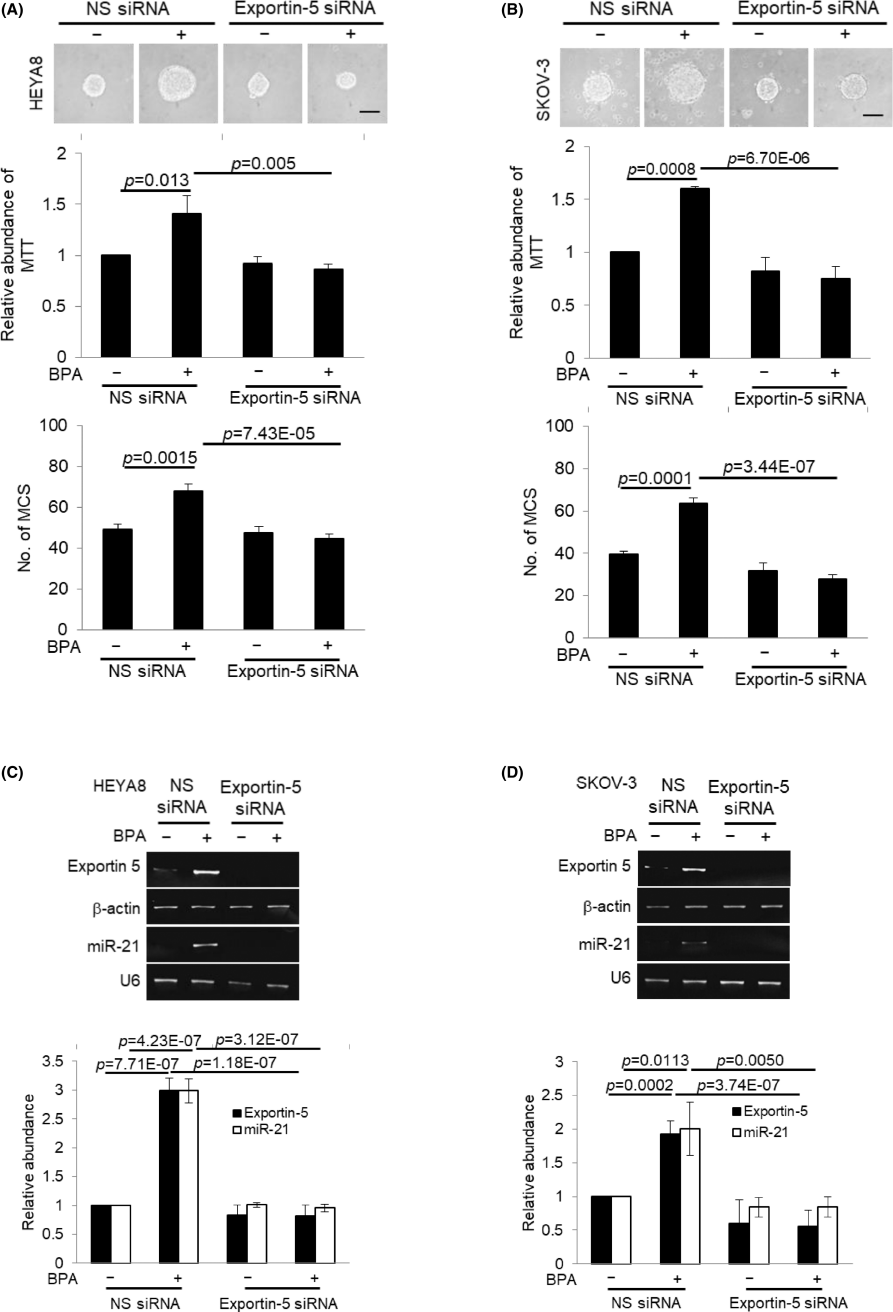
Silencing of Exportin‐5 abolishes BPA‐induced MCS formation. (A) HEYA8 and (B) SKOV‐3 were cultured with BPA at 0, 10, 50 and 100 nM in non‐adherent culture dish for 72 h. The number of tumour spheres generated was photographed, quantified by MTT assay and counted. (C) HEYA8 and (D) SKOV‐3 transfected with nonspecific (NS) siRNA or Exportin‐5 siRNA were cultured in non‐adherent culture dish for 72 h. Total RNA was extracted and reverse transcription‐PCR was performed using GPR30, Exportin‐5 and miR‐21 sequence‐specific primers. Experiments were repeated three times and results are presented as the mean ± SD and were analysed using two‐way anova.

**FIGURE 6:**
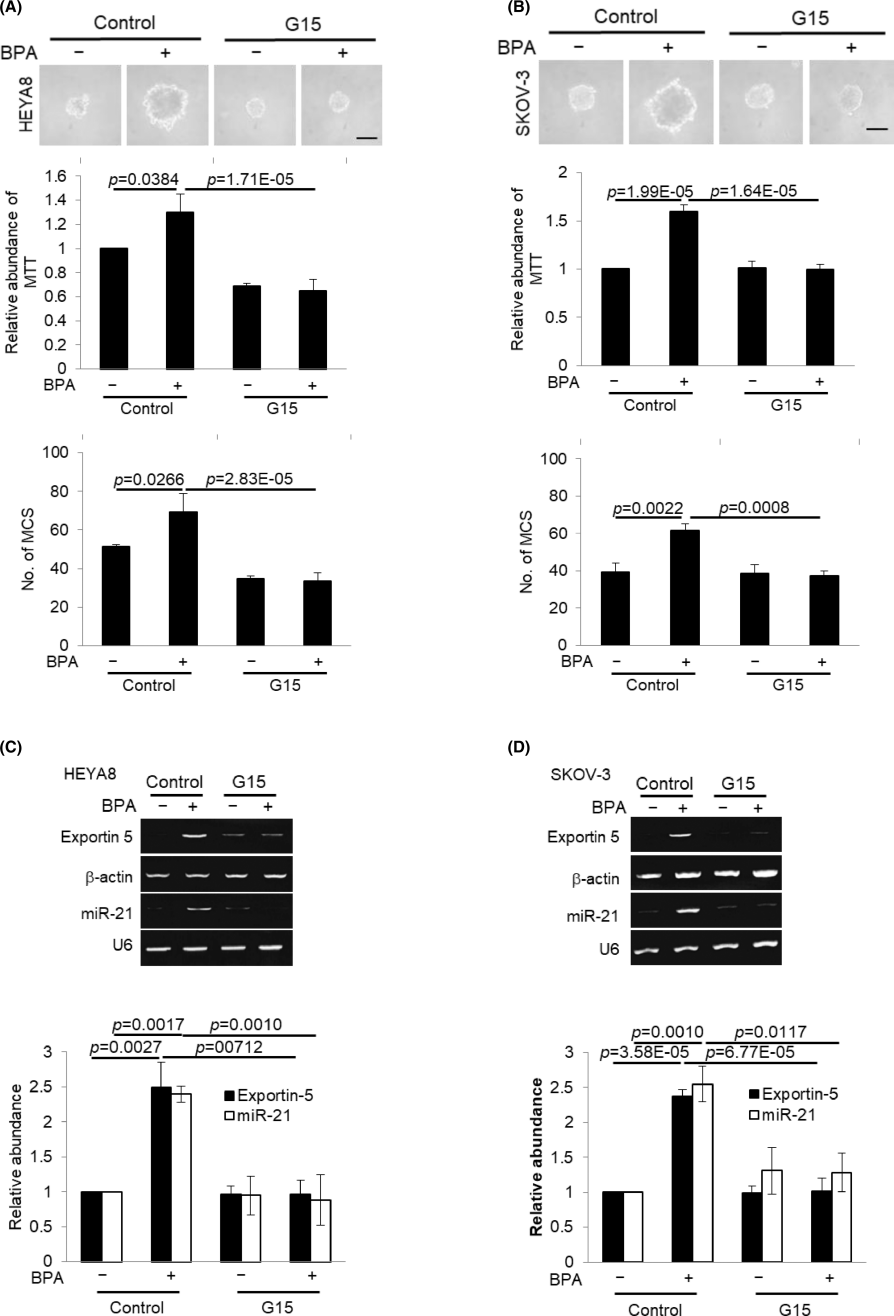
The use of GPR30 antagonist G15 abolishes BPA‐induced MCS formation. (**A**) HEYA8 and (B) SKOV‐3 were cultured with BPA at 0 and 10 nM in non‐adherent culture dish for 72 h with G15 or DMSO as the control. The number of tumour spheres generated was photographed, quantified by MTT assay and counted. (C) HEYA8 and (D) SKOV‐3 were cultured with BPA at 0 and 10 nM in non‐adherent culture dish for 72 h with G15 or DMSO as the control. Total RNA was extracted and reverse transcription‐PCR was performed using Exportin‐5 and miR‐21 sequence‐specific primers. The signal intensities were quantified by densitometric analysis and the amount was normalized for the amount of β‐Actin or U6 respectively. Bar = 100 μm. Experiments were repeated three times and results are presented as the mean ± SD and were analysed using two‐way anova.

**FIGURE 7:**
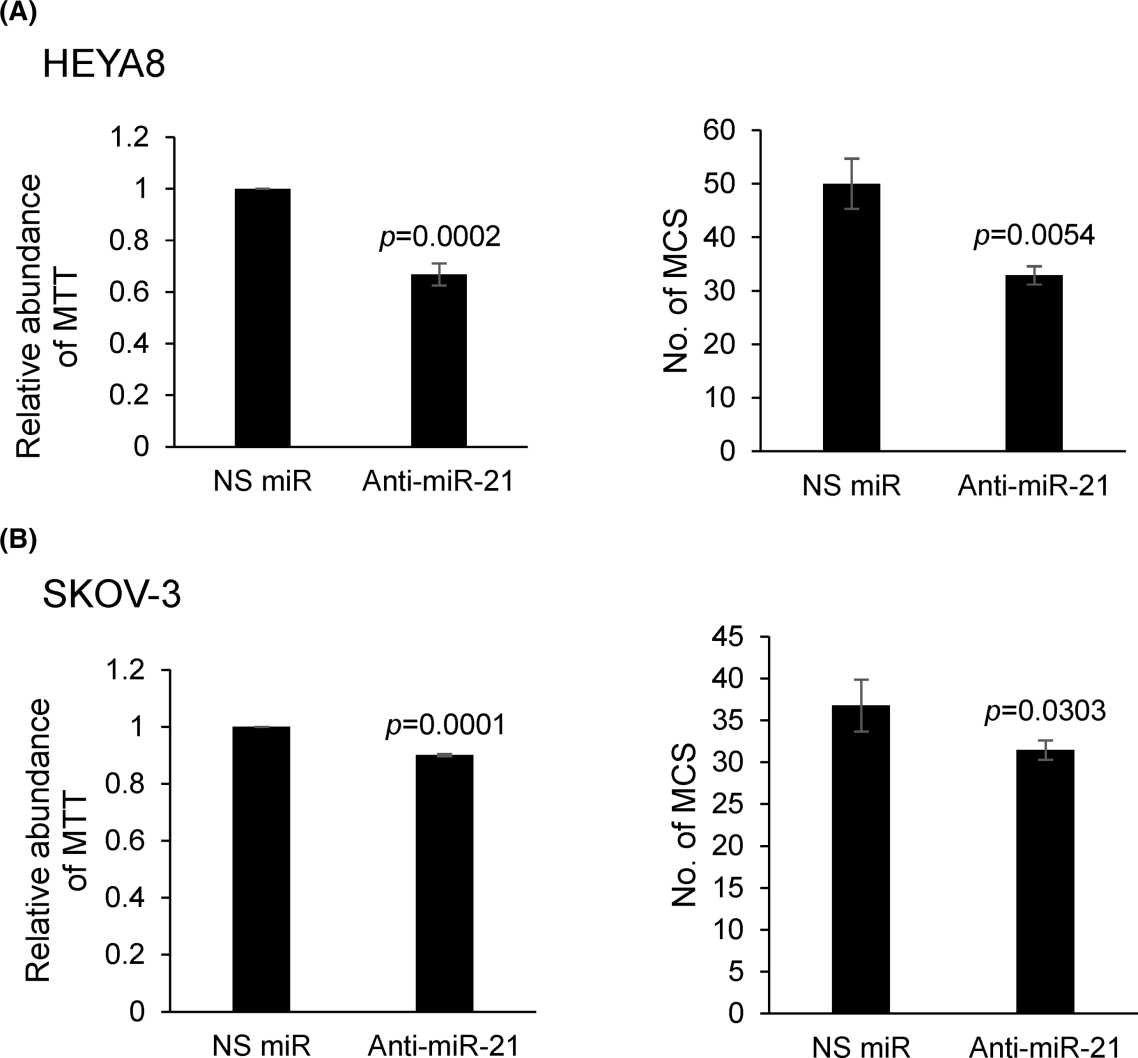
Anti‐miR21 inhibits CSC viability and spheroid formation ability. (A) HEYA8 and (B) SKOV3 cells were treated with anti‐miR21 to a working concentration of 20 nM for 72 h with NS miR as control. The number of tumour spheres generated quantified by MTT assay and counted. Experiments were repeated three times and results are presented as the mean ± SD and were analysed using unpaired student's *t* test.

To examine the consequences of the increase in Exportin‐5 expression, we assessed changes in levels of mature miRNAs, which has been previously implicated in the aggressiveness of ovarian carcinoma.^23^, ^24^ BPA led to a significant increase in oncomiR, miR‐21 (Figure 4C,D). As shown in Figure 5A,B, silencing of Exportin‐5 led to the repression of metastatic cancer spheroid (MCS) formation and growth resulting from BPA, as well is its target gene miR‐21 (Figure 5C,D), indicating that Exportin‐5 is necessary for the BPA‐mediated miRNA expression and promotion of MCS. G15 was identified as a GPR30 selective antagonist.^25^, ^26^ As shown, G15 significantly suppressed both spheroid forming and growth of HEYA8 and SKOV‐3 at 20 nM (Figure 6A,B). Furthermore, G15 treatment decreased the expression of Exportin‐5 and miR‐21 in both cell lines (Figure 6C,D). The spheroid formation ability and cell viability were also decreased upon blocking miR‐21 with the anti‐miR‐21 (Figure 7A,B).

## 4 DISCUSSION

Here we show for the first time that environmental factor BPA is essential to generate and maintain the CSC subpopulation in ER‐negative ovarian cancer via a miRNA biogenesis pathway at environmental relevant level. This may help to explain the significant cancer‐promoting properties of BPA with low exposure. It is noteworthy that cancer cells are plastic and have been shown to dynamically switch from non‐CSC to CSC. For example, such phenotypic plasticity by non‐CSCs has recently been shown in melanoma and other tumour types.^27^, ^28^ The unstable traits observed in cancer cells may reflect a high degree of flexibility that favours tumour adaption to variations to facilitate tumour progression. This process is regulated by both intrinsic and extrinsic factors. One challenge encountered in studies of cancer and the environment is the lack of reliable exposure. Our results may provide a probable explanation as to the inconsistent findings of BPA in ovarian cancer. Moreover, the concentrations of BPA used in our experiments are within the physiological levels of 0.2–18.9 ng/mL in the plasma commonly seen in adults or fetus serum, our findings are particularly relevant to human exposure levels.^29^, ^30^, ^31^

The effects of BPA on mitogenesis to date thus far have used the ER‐positive ovarian cancer cells, of which it has been reported that 25%–86% of ovarian cancer have ER expression.^32^ For example, Park et al. observed increased BG‐1 cell proliferation in response to BPA.^10^ Ptak et al. further confirmed the prosurvival effect of BPA using OVCAR‐3.^33^ The SKOV‐3 has been used as a model for ER‐negative ovarian cancer since a 32‐bp deletion in exon 1 of ERα transcript has been found, and thus it is ER‐positive but insensitive to E_2_ with respect to cell proliferation and induction of gene expression. Interestingly, we also observed an effect of BPA on this ER‐defective SKOV‐3 cells, raising the intriguing possibility that ERα may not mediate the carcinogenic effects of BPA. This is also in line with the fact that BPA binds to ERs only at micromolar range, suggesting that BPA may act through nonclassical ERs at low doses. We also observed its ability to enhance CSC formation and expansion in an ER‐independent manner. Nonclassical ERs include ERRs and GPR30. Importantly, GPR30 mRNA level was significantly higher in ovarian tumours relative to normal ovarian surface epithelial cells and GPR30 expression predicts poor survival for ovarian cancer patients.^34^

Our data indicate an important role for miRNAs in the BPA‐conferred CSC formation and expansion. Although different mechanisms may be involved, the stimulation of oncomiR biogenesis is of particular interest because it clearly supports CSC survival as well as self‐renewal and tumorigenicity. Moreover, differential expression of miRNA and the downstream target genes resulted from the deregulation of miRNA biogenesis machinery may also explain why different mechanisms have been reported in mediating the effects of BPA. Altered miRNA biogenesis is associated with multiple poor prognostic factors in human cancers, including ovarian cancer.^35^ In several model systems, Exportin‐5 has been shown to stimulate cell growth. Apart from being an exporter of double‐stranded RNA out of the nucleus to the cytoplasm, Exportin‐5 can also post‐transcriptionally regulate the expression of Dicer showing its importance in the miRNA biogenesis machinery. Global change in miRNA level induced by Exportin‐5 expression has been previously reported.^36^ Several mechanisms have been described as potential regulators of Exportin‐5 level including the modulation of mRNA stability through PI3K signalling. Exportin‐5 expression has also been reported to be regulated by epigenetic cofactors.^37^ Whether BPA uses these or other mechanisms driving this aggressive phenotype awaits further investigation.

This is the first report that the endocrine disrupter BPA has the capacity to enhance the formation and expansion of CSCs in ER‐negative ovarian cancer cells. Importantly, unlike some previous studies that have used high micromolar doses of BPA, we have used low nanomolar doses, which are relevant to human exposure levels.^4^, ^5^ Moreover, BPA does not appear to mediate its effect through the classical ERs in ovarian cancer cells. Rather, nonclassical GPR30 could serve as putative receptor that sensitize BPA at nanomolar level. In support, ER‐independent IGF‐1R, TGF‐β, JAK/STAT3, MAPK/ERK and PI3K/Akt were also previously reported to mediate BPA actions in ovarian cancer cells.^38^ VEGR was found to be stimulated in breast cancer.^39^ AR could essentially mediate the actions of BPA in advanced prostate adenocarcinomas.^40^ We also provide evidence that a new mechanism in global miRNA regulation is involved in this process, which may well explain the observation of its significant tumour‐promoting effects which could provide important insights for setting up guidelines for limiting human exposure.

## AUTHOR CONTRIBUTIONS

**Sophia S. N. Lam:** Conceptualization (lead); data curation (equal); formal analysis (equal); writing – original draft (equal). **Zeyu Shi:** Writing – original draft (lead); writing – review and editing (lead). **Carman K. M. Ip:** Conceptualization (equal); data curation (equal); formal analysis (equal); methodology (equal); writing – original draft (equal). **Chris K. C. Wong:** Resources (equal); writing – review and editing (equal). **Alice S. T. Wong:** Funding acquisition (lead); writing – review and editing (equal).

## FUNDING INFORMATION

This work was supported by the HKU CRCG Grant and Strategic Research Theme Fund on Cancer. We also acknowledge the funding support from “Laboratory for Synthetic Chemistry and Chemical Biology” under the Health@InnoHK Program launched by Innovation and Technology Commission, The Government of Hong Kong Specical Administrative Region of the People's Republic of China. A.S.T.W. is a recipient of the Croucher Senior Research Fellowship.

## CONFILCT OF INTEREST STATEMENT

The authors declare no conflict of interest.

## Data Availability

Data available on request from the authors.
